# Draft Genome Sequence of *Enterococcus faecalis* MB5259

**DOI:** 10.1128/genomeA.00634-14

**Published:** 2014-06-19

**Authors:** Andrey V. Karlyshev, Joris Robyn, Geertrui Rasschaert, Marc Heyndrickx

**Affiliations:** a School of Life Sciences, Faculty of Science, Engineering, and Computing, Kingston University, Kingston upon Thames, United Kingdom; b Institute for Agricultural and Fisheries Research, Technology and Food Science Unit, Melle, Belgium; c Department of Pathology, Bacteriology, and Poultry Diseases, Ghent University, Merelbeke, Belgium

## Abstract

In this study, we present a draft genome sequence of *Enterococcus faecalis* MB5259, a promising probiotic strain. The identified differences and common features between this strain and reference strains will assist in better understanding the mechanism of antibacterial action and in developing novel probiotics.

## GENOME ANNOUNCEMENT

*Campylobacter jejuni* is the major cause of gastrointestinal infections worldwide. One promising intervention strategy to combat the disease caused by this pathogen is the development of antibacterials based on probiotics. Our previous large-scale screening involving 1,250 bacterial strains identified a particular strain of *Enterococcus faecalis*, MB5259, possessing superior antibacterial activity against *C. jejuni in vitro*, in which a classical bacteriocin is probably not involved (1). Although *in vivo* trials revealed no protection against *C. jejuni* colonization (2), the strain can possibly delay colonization (3). The aim of this study was to produce a draft genome sequence of strain MB5259, which would assist in understanding of the mechanism of its antibacterial action.

The sequencing data were generated using an Ion Torrent PGM with 314v2 chip and an Ion PGM Sequencing 400 kit. The data were collected from two runs with median sequencing read sizes of 316 nucleotides (nt) and 338 nt. The Ion Torrent *de novo* assembler plugin generated 46 contigs ranging between 1 kb and 559 kb in size. Read mapping to a reference *E. faecalis* Symbioflor 1 genome (4, 5) and generation of consensus sequences using CLC Genomics Workbench allowed the reduction of the number of contigs to 38, ranging between 1 and 717 kb, with *N*_50_ = 411 kb. The genome size (3,106,425 nt with coverage of 72.16×) and G+C content (37.5%) were in agreement with published data for other strains of this species (2.74 to 3.30 Mb and 37.4 to 37.8%, respectively).

Analysis of unmapped reads revealed the genes present in the test strain but absent in the reference strain. One of these genes encodes gelatinase (coccolysin) GelE, a secreted peptidase found in some other strains of *E. faecalis*. GelE controls cellular chain length (6) and may also be involved in immune evasion via degradation of some components of the complement system (7). Another gene present in the test strain but not in the reference strain was the one encoding a ferro-lyase.

Read mapping onto the Symbioflor 1 genome also revealed the genes present in the reference but not in the test genome. Among these were a number of hypothetical genes and the genes encoding transposases, DNA-binding response regulators, and sensor histidine kinases, as well as a large gene cluster (ca. 40 kb) containing polysaccharide biosynthesis-related genes. The strains shared genes encoding colicin V production family protein (100% identity) and two collagen-specific adhesins, as well as Zeta toxin, involved in bacterial dormancy.

Mapping of reads onto available sequences of *E. faecalis* plasmids revealed similarity to the plasmids pTEF1 and pTEF2 but not to pTEF3 of strain V583. Instead, a 5.1-kb plasmid identical to EF62pA from strain 62 was found.

Genome annotation using the RAST server (8) reported 3,056 protein-coding sequences. Among the other features, the genes responsible for resistance to beta-lactam antibiotics, vancomycin, and fluoroquinolones were detected.

Further studies on comparative analysis of the genomes of various *E. faecalis* strains may assist in the development of novel recombinant probiotic strains.

### Nucleotide sequence accession numbers.

This whole-genome shotgun project has been deposited at DDBJ/EMBL/GenBank under the accession number JMEC00000000. The version described in this paper is version JMEC01000000.

## References

[1] Robyn J Rasschaert G Messens W Pasmans F Heyndrickx M 2012 Screening for lactic acid bacteria capable of inhibiting *Campylobacter jejuni* in *in vitro* simulations of the broiler chicken caecal environment. Benef. Microbes 3:299–308. 10.3920/BM2012.0021 23234730

[2] Robyn J Rasschaert G Hermans D Pasmans F Heyndrickx M 2013 In vivo broiler experiments to assess anti-*Campylobacter jejuni* activity of a live *Enterococcus faecalis* strain. Poult. Sci. 92:265–271. 10.3382/ps.2012-02712 23243257

[3] Robyn J 2013 *In vitro* and *in vivo* evaluation of measures to control *Campylobacter jejuni* in broilers. Ph.D. thesis. Ghent University, Merelbeke, Belgium

[4] Fritzenwanker M Kuenne C Billion A Hain T Zimmermann K Goesmann A Chakraborty T Domann E 2013 Complete genome sequence of the probiotic *Enterococcus faecalis* Symbioflor 1 clone DSM 16431. Genome Announc. 1(1):e00165-12. 10.1128/genomeA.00165-12 23405346 PMC3569346

[5] Domann E Hain T Ghai R Billion A Kuenne C Zimmermann K Chakraborty T 2007 Comparative genomic analysis for the presence of potential enterococcal virulence factors in the probiotic *Enterococcus faecalis* strain Symbioflor 1. Int. J. Med. Microbiol. 297:533–539. 10.1016/j.ijmm.2007.02.008 17466591

[6] Waters CM Antiporta MH Murray BE Dunny GM 2003 Role of the *Enterococcus faecalis* GelE protease in determination of cellular chain length, supernatant pheromone levels, and degradation of fibrin and misfolded surface proteins. J. Bacteriol. 185:3613–3623. 10.1128/JB.185.12.3613-3623.2003 12775699 PMC156229

[7] Park SY Shin YP Kim CH Park HJ Seong YS Kim BS Seo SJ Lee IH 2008 Immune evasion of *Enterococcus faecalis* by an extracellular gelatinase that cleaves C3 and iC3b. J. Immunol. 181:6328–6336. 10.4049/jimmunol.181.9.6328 18941224

[8] Aziz RK Bartels D Best AA DeJongh M Disz T Edwards RA Formsma K Gerdes S Glass EM Kubal M Meyer F Olsen GJ Olson R Osterman AL Overbeek RA McNeil LK Paarmann D Paczian T Parrello B Pusch GD Reich C Stevens R Vassieva O Vonstein V Wilke A Zagnitko O 2008 The RAST server: Rapid annotations using subsystems technology. BMC Genomics 9:75. 10.1186/1471-2164-9-75 18261238 PMC2265698

